# Efficacy and cost-effectiveness of two online interventions for children and adolescents at risk for depression (E.motion trial): study protocol for a randomized controlled trial within the ProHEAD consortium

**DOI:** 10.1186/s13063-018-3156-8

**Published:** 2019-01-15

**Authors:** Sabrina Baldofski, Elisabeth Kohls, Stephanie Bauer, Katja Becker, Sally Bilic, Heike Eschenbeck, Michael Kaess, Markus Moessner, Hans Joachim Salize, Silke Diestelkamp, Elke Voß, Christine Rummel-Kluge, the ProHEAD consortium, Michael Kaess, Stephanie Bauer, Rainer Thomasius, Christine Rummel-Kluge, Heike Eschenbeck, Hans-Joachim Salize, Katja Becker, Katja Bertsch, Sally Bilic, Romuald Brunner, Johannes Feldhege, Christina Gallinat, Sabine C. Herpertz, Julian Koenig, Sophia Lustig, Markus Moessner, Fikret Özer, Peter Parzer, Franz Resch, Sabrina Ritter, Jens Spinner, Silke Diestelkamp, Kristina Wille, Sabrina Baldofski, Elisabeth Kohls, Lina-Jolien Peter, Vera Gillé, Hanna Hofmann, Laya Lehner, Elke Voß, Jens Pfeiffer, Alisa Samel

**Affiliations:** 1 Department of Psychiatry and Psychotherapy, Medical Faculty, University Leipzig, Semmelweisstraße 10, 04103 Leipzig, Germany; 2 Center for Psychotherapy Research, University Hospital Heidelberg, Heidelberg, Germany; 3 Department of Child and Adolescent Psychiatry, Psychosomatics and Psychotherapy, University Hospital of Marburg and Philipps-University Marburg, Marburg, Germany; 4 Marburg Center for Mind, Brain and Behavior (MCMBB), Philipps-University Marburg, Marburg, Germany; 5 Department of Psychology, University of Education Schwäbisch Gmünd, Schwäbisch Gmünd, Germany; 6 Department of Child and Adolescent Psychiatry, Centre for Psychosocial Medicine, University Hospital Heidelberg, Heidelberg, Germany; 7 University Hospital of Child and Adolescent Psychiatry and Psychotherapy, University of Bern, Bern, Switzerland; 8 Mental Health Services Research Group, Central Institute of Mental Health, Medical Faculty Mannheim/Heidelberg University, Mannheim, Germany; 9 German Center for Addiction Research in Childhood and Adolescence (DZSKJ), University Medical Center Hamburg-Eppendorf, Hamburg, Germany

**Keywords:** Depression, Depressive symptoms, Children, Adolescents, Adolescence, Prevention, Internet-based, Psychological intervention, Self-management, ProHEAD

## Abstract

**Background:**

Depression is a serious mental health problem and is common in children and adolescents. Online interventions are promising in overcoming the widespread undertreatment of depression and in improving the help-seeking behavior in children and adolescents.

**Methods:**

The multicentre, randomized controlled E.motion trial is part of the German ProHEAD consortium (Promoting Help-seeking using E-technology for ADolescents). The objective of the trial is to investigate the efficacy and cost-effectiveness of two online interventions to reduce depressive symptomatology in high-risk children and adolescents with subsyndromal symptoms of depression in comparison to an active control group. Participants will be randomized to one of three conditions: (1) Intervention 1, a clinician-guided self-management program (iFightDepression®); (2) Intervention 2, a clinician-guided group chat intervention; and (3) Control intervention, a psycho-educational website on depressive symptoms. Interventions last six weeks. In total, *N* = 363 children and adolescents aged ≥ 12 years with Patient Health Questionnaire-9 modified for Adolescents (PHQ-A) scores in the range of 5–9 will be recruited at five study sites across Germany. Online questionnaires will be administered before onset of the intervention, at the end of the intervention, and at the six-month follow-up. Further, children and adolescents will participate in the baseline screening and the one- and two-year school-based follow-up assessments integrated in the ProHEAD consortium. The primary endpoint is depression symptomatology at the end of intervention as measured by the PHQ-A score. Secondary outcomes include depression symptomatology at all follow-ups, help-seeking attitudes, and actual face-to-face help-seeking, adherence to and satisfaction with the interventions, depression stigma, and utilization and cost of interventions.

**Discussion:**

This study represents the first randomized controlled trial (RCT) investigating efficacy and cost-effectiveness of two online interventions in children and adolescents aged ≥ 12 years at risk for depression. It aims to provide a better understanding of the help-seeking behavior of children and adolescents, potential benefits of E-mental health interventions for this age group, and new insights into so far understudied aspects of E-mental health programs, such as potential negative effects of online interventions. This knowledge will be used to tailor and improve future help offers and programs for children and adolescents and ways of treatment allocation.

**Trial registration:**

German Register for Clinical Trials (DRKS), DRKS00014668. Registered on 4 May 2018.

International trial registration took place through the “international clinical trials registry platform” with the secondary ID S-086/2018.

**Electronic supplementary material:**

The online version of this article (10.1186/s13063-018-3156-8) contains supplementary material, which is available to authorized users.

## Background

Depressive disorders are severe mental illnesses with overall prevalence rates, measured from point prevalence up to 12-month prevalence, in the range of 2.6–19.4% among children and adolescents (C&A) [1–7]. Rates rise in adolescence compared to childhood [8] and show a stronger female preponderance in adolescents [2, 6]. Depression in C&A has a marked negative impact regarding poor academic performance and social dysfunction and predicts adverse mental health outcomes such as recurrence of depressive symptoms, substance abuse, self-harm, suicidal ideation, and attempted and completed suicide [9–11]. Due to the high burden of depression for both personal and societal areas, early intervention and prevention of its onset are of utmost importance [12, 13]. Targeting subthreshold depression in particular is of enormous potential as subthreshold depression in adolescents is a substantial risk factor for the onset of major depression [14]. However, currently the vast majority of C&A with subthreshold depressive symptoms does not seek professional help and does not receive professional support. Barriers to help-seeking include poor mental health literacy, shame and stigmatization related to depression [15], but also symptoms of depression itself, such as feelings of worthlessness or guilt. Internet-based interventions present a promising approach to overcome these barriers, especially as various studies have shown that a high percentage of C&A use the Internet for health-related purposes (e. g. seeking health-related information and support communities; [16]). Internet-based interventions provide low-threshold access to evidence-based information, self-help tools, peer support, and professional counseling and could thus help in reaching target populations who might otherwise not seek treatment.

The development of online-delivered interventions and the use of smartphone systems and apps for monitoring and treatment of depression has increased enormously during recent years [17]. Therapist-delivered online cognitive behavioral therapy (CBT) for depression has been shown to be both effective and cost-effective [18]. In addition, various guided and unguided Internet-based interventions for adults with depression have been developed and their efficacy has been shown [12]. Specifically, self-management and self-help reduce the burden of sub-threshold and full-syndrome forms of depression in adults [19]. Comparisons of Internet-based interventions for depression with face-to-face treatment did not indicate differences in efficacy between the different treatment formats [20].

Despite the clear benefits of Internet-based interventions for depression in adults, there is a lack of studies specifically focusing on C&A [21, 22]. Several pilot trials have been published recently, providing preliminary results for the effectiveness of Internet-based interventions for the treatment of depression in C&A. These include, among others, an Internet-based intervention for at-risk of suicide school students, which has resulted in reduced suicidal ideation, hopelessness, and depressive symptoms [23], a randomized controlled trial (RCT) on a spirituality informed e-mental health tool as an intervention for major depressive disorder in adolescents and young adults [24], and a RCT on a school-based CBT program, which resulted in significant decreases in depression symptomatology and suicidality [25]. Another self-directed Internet-based intervention for depression was delivered as part of the high school curriculum and resulted in a reduction of depressive symptoms in adolescent girls [26].

While most programs include online information, exercises, and questionnaires accessible to the participants, chat treatments for C&A at risk for depression have not been studied well so far. Two randomized trials in adolescents and young people with depressive symptoms investigating the effectiveness of an Internet-based, solution-focused, brief chat treatment and an Internet-based chat treatment based on principles of CBT, respectively, showed positive effects of the respective treatment regarding a reduction of depressive symptoms in comparison to a wait-list control condition [22, 27].

In summary, there is preliminary evidence for the effectiveness of Internet-based interventions in C&A at risk for depression. However, most of the identified trials had either limited results, only short-term follow-up periods (often due to waitlist control conditions), or were self-guided. Studies with longer-term follow-up and guidance are currently lacking [22]. To address the gaps identified in the literature, the E.motion trial will be conducted to investigate the efficacy and cost-effectiveness of two different online interventions to reduce depressive symptomatology in high-risk C&A aged ≥ 12 years with subsyndromal symptoms of depression.

## Methods/Design

### Design

The E.motion trial is a multicentre, three-arm RCT comparing two online interventions with an active control group. Participants will be randomized to one of three conditions, each with a duration of six weeks: (1) Intervention 1, a clinician-guided self-management program (iFightDepression®); (2) Intervention 2, a clinician-guided group chat intervention; and (3) Control intervention, a psycho-educational website on depressive symptoms.

### Recruitment and study procedures

This trial is part of the ProHEAD consortium (Promoting Help-seeking using E-technology for ADolescents) aiming to assess new access pathways for prevalent mental health problems in C&A. Within the ProHEAD consortium, C&A with mental health problems and/or high risk for mental health problems will be allocated to one of five RCTs [28–31]. A large-scale school-based sample of *N* = 15,000 C&A aged ≥ 12 years attending school grades 6–13 will be recruited within the ProHEAD consortium in five urban areas geographically distributed across Germany (Hamburg, Heidelberg, Leipzig, Marburg, Schwäbisch Gmünd). The ProHEAD consortium includes online assessments of mental health problems and health-risk behaviors (i.e. general mental health problems, eating disorder symptoms, alcohol use, and depressive symptoms) at baseline and at two annual follow-ups. All assessments within the ProHEAD consortium will be administered through on-site visits in the school classes and C&A will complete the questionnaires in the respective school’s local computer rooms (for further details on study procedures within the ProHEAD consortium, see study protocol by Kaess et al., published in this special issue).

Written informed consent will be obtained from all participating C&A and their legal guardians before administering baseline screening assessments. Participants will be informed that after completing the baseline screening, they will receive an invitation to participate in one out of five fully remote Internet-based intervention trials (group 1, general mental health problems; group 2, eating disorder symptoms; group 3, at-risk alcohol use; group 4, depressive symptoms, E.motion trial; group 5, no mental health problems, prevention trial). Within each of these RCTs, different intervention and prevention programs, respectively, will be compared and the allocation to a specific RCT will be based on meeting pre-defined cut-off scores in the baseline screening.

**Fig. 1:**
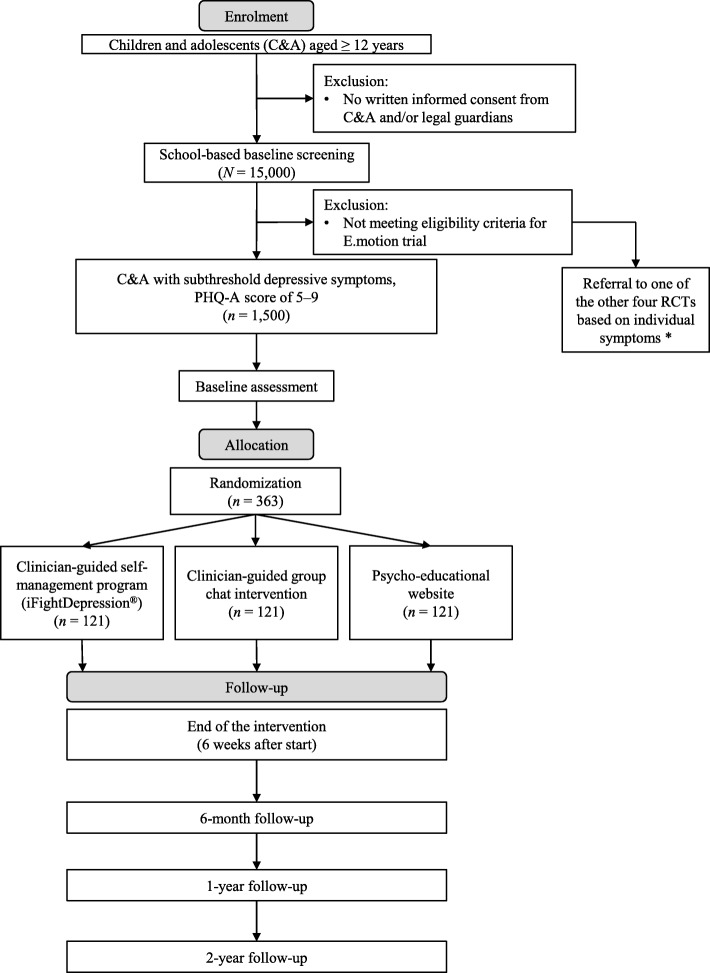
Trial flow *diagram*. Assessments within the E.motion trial will be conducted at baseline, at the end of intervention (six weeks after onset of intervention), and at six-month follow-up. Further, participants will complete the school-based baseline screening and one- and two-year school-based follow-up assessments integrated in the ProHEAD consortium. * For further details on other RCTs within the ProHEAD consortium, see other study protocols published in this special issue. PHQ-A Patient Health Questionnaire-9 modified for Adolescents

**Fig. 2:**
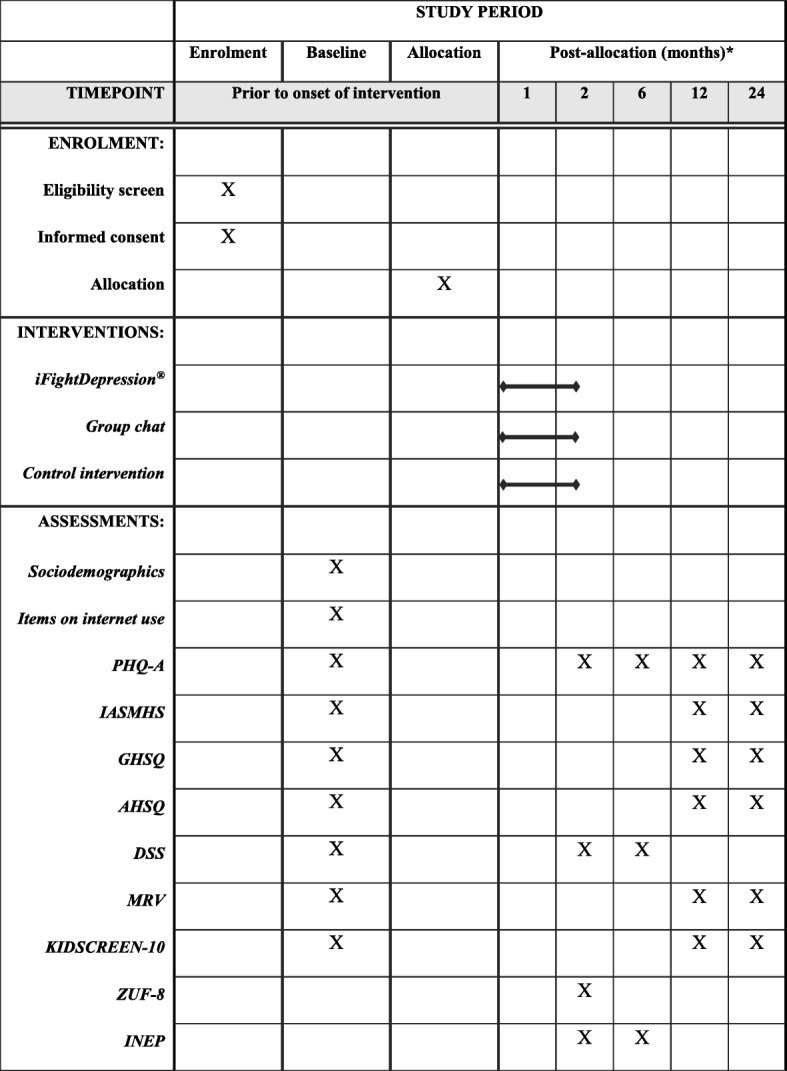
Schedule of enrolment, interventions, and assessments. * Assessments within the E.motion trial will be conducted at the end of intervention (six weeks after onset of intervention) and at six-month follow-up. Further, participants will complete the one- and two-year school-based follow-up assessments integrated in the ProHEAD consortium. PHQ-A Patient Health Questionnaire-9 modified for Adolescents, IASMHS Inventory of Attitudes Toward Seeking Mental Health Services, GHSQ General Help-Seeking Questionnaire, AHSQ Actual Help-Seeking Questionnaire, DSS Depression Stigma Scale, MRV Mannheimer Modul zum Ressourcenverbrauch, KIDSCREEN-10 Health-related quality of life measure for children and adolescents, ZUF-8 German version of the Client Satisfaction Questionnaire, INEP Inventory for the Assessment of Negative Effects of Psychotherapy

Participants for the E.motion trial will be recruited from the ProHEAD consortium. Following the baseline screening within the ProHEAD consortium, an estimated number of *N* = 1500 C&A reporting subthreshold depressive symptoms and meeting eligibility criteria for the E.motion trial will be invited to participate in the trial. In the case of participation, these C&A will be randomized to one of the three treatment arms, as illustrated in Fig. 1. Participants not meeting eligibility criteria will be included in one of the other four RCTs within the ProHEAD consortium (see information on eligibility criteria below). A schedule of enrolment, interventions, and assessments is included in Fig. 2 and a Standard Protocol Items: Recommendations for Intervention Trials (SPIRIT) Checklist is provided in Additional file 1.

### Eligibility criteria

All eligibility criteria will be assessed through the baseline screening of the ProHEAD consortium. C&A aged ≥ 12 years and reporting current subthreshold depressive symptoms will be included in the trial. Subthreshold depressive symptoms will be operationalized with the Patient Health Questionnaire-9 modified for Adolescents (PHQ-A) [32, 33], with scores in the range of 5–9. Inclusion criteria further comprise sufficient German language skills and Internet access. There are no restrictions for participants for engaging in additional (depression-related) interventions or treatments.

Participants reporting either other mental health problems (general mental health problems, eating disorder symptoms, or alcohol misuse) or no clinically relevant levels of psychopathology will be allocated based on their specific symptom profiles to one of the other four RCTs within the ProHEAD consortium. Other current mental health problems will be operationalized as scores above the clinical cut-off in one or more of the following questionnaires: Strengths and Difficulties Questionnaire (SDQ) [34]; Short Evaluation of Eating Disorders (SEED) [35]; Weight Concerns Scale (WCS) [36]; Car, Relax, Alone, Forget, Friends, Trouble (CRAFFT-d) [37]; and Alcohol Use Disorders Identification Test (AUDIT) [38]. Participants meeting inclusion criteria for more than one RCT will be randomly allocated to one of the RCTs for which they are eligible.

### Assessments

All interventions will have a duration of six weeks for the individual participant. Assessments specific to the E.motion trial will be conducted before the onset of the intervention period, at the end of the intervention (six weeks after start of intervention), and at the six-month follow-up. Further, C&A will participate in the baseline screening and the regular one- and two-year school-based follow-up assessments integrated in the ProHEAD consortium, to track long-term outcome of the interventions (see Fig. 2). All assessments will consist of self-administered questionnaires and will be carried out online using secure links sent via automated e-mails.

### Randomization

Following the school-based baseline screening within the ProHEAD consortium, participants eligible for the E.motion trial will receive an e-mail including information about the trial and access details for the baseline assessment within the E.motion trial. After completing this assessment, participants will be randomized with equal probability (in a 1:1:1 ratio) to one of the three intervention groups. The assignment to the three conditions will be randomized, stratified for gender and school type. Randomization will be conducted externally and follow a permutated block design. Following randomization, participants will be contacted again via e-mail to be informed about their allocation to the respective intervention group and receive access details for the specific online programs. Blinding is not possible as the study assistants will be involved in delivering the interventions. However, the risk of a potential bias is considered minimal as the study outcome variables are exclusively based on self-report. For all treatment arms, the intervention will be discontinued at any time for participants who withdraw consent to participate in the trial.

### Interventions

#### Intervention 1: iFightDepression® (iFD®) tool

The first intervention group will complete the iFightDepression® (iFD®) tool, which is an online, clinician-guided, self-management program that aims to help individuals with mild to moderate depression to self-manage their symptoms (tool accessible at https://tool.ifightdepression.com) [39]. The tool consists of six core modules related to behavioral activation, sleep and mood monitoring, and cognitive restructuring (“Thinking, feeling and doing,” “Planning and doing things that you enjoy,” “Getting things done,” “Recognizing negative thoughts,” “Changing negative thoughts,” and “Sleep and depression”) and three optional modules related to psychosocial issues and healthy lifestyle habits (“Healthy lifestyle,” “Relationships,” and “Social anxiety”). Each module comprises written information, worksheets (online or printable), exercises, and a voluntary mood rating using the PHQ-A with a graphical output display. In addition, emergency contact material is provided. Participants will be asked to use the tool for six weeks on a regular basis and complete one workshop per week. The self-management program has a total duration of six weeks and participants will be guided by a trained clinical psychologist giving administrative and motivational support and exploring how well participants were able to integrate the exercises into their daily routine.

Two versions of the iFD® tool (one for adults and one for young people) were developed within the EU-funded project PREDI-NU [39]. Previous research confirmed the feasibility of the tool [39, 40]. Oehler C, Görges F, Böttger D, Hug J, Koburger N, Kohls E, et al: Efficacy of an internet-based self-management intervention for adult primary care patients with mild and moderate depression or dysthymia – a study protocol, submitted. Within the E.motion trial, the tailored version of the tool for young people, incorporating less formal language and additional age-appropriate modules on relationships and social anxiety, will be used. iFD® was developed based on existing evidence, best practice recommendations, and user and expert consensus. The tool is free to use and is intended to help individuals self-manage their symptoms of depression and to promote recovery. It is based on principles of CBT, which has been proven to be effective in treating depression [41, 42]. Associated worksheets and exercises encourage users of the tool to practice and consolidate new skills and to promote self-monitoring. The tool is currently available in 11 languages.

#### Intervention 2: Group chat

The second intervention group will receive a clinician-guided, online, group chat intervention based on a cognitive-behavioral approach. The intervention will address the following core elements: (1) information about depressive symptoms; (2) diagnosis of depression and etiological factors; (3) treatment options: psychotherapy; (4) treatment options: medication; (5) social support and dealing with stigmatization in the context of depression; and (6) emergency strategies and help seeking. The intervention will consist of six 90-min group chat sessions, which will be held once per week. Sessions will be scheduled at fixed times and a secured chat room will be specifically arranged for the sessions. The group size will be 6–10 participants, depending on recruitment and attrition rates. Groups will be “open,” i.e. participants can join at any time during the intervention period. Participants will communicate via written messages, allowing a synchronous communication in real time. Participation in the chat will be under self-chosen pseudonyms to ensure confidentiality.

The chats will be moderated by a trained clinical psychologist and the intervention will be manualized to maximize treatment adherence. The psychologists delivering the intervention are experienced in individual and group treatment of persons with depression and depressive symptoms, being aware of the medical consequences of depressive symptoms, potential suicidal ideation, and self-harm. These aspects will be assessed regularly during chat sessions. Each chat session will be focused on one of the six core elements as mentioned above, whereas the specific chat agenda will be set at the beginning of each session and will be based on the current questions and needs of each participant. Sessions will be used to provide information on different aspects of depression and to discuss and answer questions within the group. A specific focus will be given to positive communication among participants and to the peer-to-peer exchange, which has been shown to be of additional help in patients with depression and other mental disorders [43–47]. Kohls E, Hug J, Stahl M, Driessen P, Roemer C, Wollschlaeger E, et al: Peer counselling in depression care: a pilot study in an inpatient setting, submitted. Chat protocols will be stored for supervision purposes.

#### Control intervention

Trial participants in the control intervention group will have access to structured online psycho-educational modules on depression (e.g. symptoms, diagnosis, treatment, medication, psychotherapy, other forms of treatment like self-help) for a duration of six weeks. There will be no therapeutic guidance or support. The participants in the control condition will be offered to inform themselves about the different aspects of depression by reading the different psycho-educational modules on their own schedule, e.g. one module per week.

### Intervention fidelity

The guiding therapists for the iFD® tool will be clinical psychologists who have qualified through completing a guidance webinar and a short test before gaining access to the tool. For the group chat, clinical psychologists will conduct the chats and will have completed a training according to the group chat intervention manual before the start of the intervention. For both interventions 1 and 2, regular supervision will be conducted by a senior psychiatrist (CRK) specializing in depression, who has been part of the project team developing the iFD® tool and has developed the manual for the clinician-guided group chat intervention.

### Outcomes and measures

#### Primary outcome

The primary outcome of the trial is depression symptomatology at the end of intervention as measured by the PHQ-A score. The depression module of the PHQ-A [32, 33] comprises nine items assessing depressive symptoms based on the diagnostic criteria for depression according to the fourth edition of the Diagnostic and Statistical Manual of Mental Disorders (DSM-IV) [48]. Respondents rate the frequency of the symptoms over the previous two weeks on a four-point Likert scale (0 = “not at all” to 3 = “nearly every day”). A sum score will be computed with higher scores indicating higher levels of depressive symptomatology. The PHQ-9 for adults has shown good reliability, validity, and sensitivity to change [49, 50].

#### Secondary outcomes

Secondary outcomes will be assessed at baseline, at the end of intervention, and at six months, one year, and two years after the start of the intervention by using the following measures: the PHQ-A [32, 33] will be used to assess depression symptomatology. Help-seeking attitudes will be assessed by the Inventory of Attitudes Toward Seeking Mental Health Services (IASMHS) [51] and actual face-to-face help-seeking will be measured with the General Help-Seeking Questionnaire (GHSQ) [52] and the Actual Help-Seeking Questionnaire (AHSQ) [53]. Adherence to the interventions (iFightDepression® and group chat) will be measured with participation rates and satisfaction with the interventions as well as possible negative effects will be assessed by the German version of the Client Satisfaction Questionnaire (ZUF-8) [54] and the Inventory for the Assessment of Negative Effects of Psychotherapy (INEP) [55], respectively. The Depression Stigma Scale (DSS) [56] will be used to assess the stigma associated with depression.

Finally, cost-effectiveness and cost-utility analyses will be conducted. Data on the cost of interventions will be compared to study outcomes to determine the incremental cost-effectiveness ratio (ICER) of interventions. The ICER is defined as the differential cost of a new treatment and treatment as usual, divided by the outcome differential of the two. Cost-utility analyses will provide information on cost per quality-adjusted life years (QALYs). QALYs are measures combining the additional life years gained by a certain healthcare intervention or program with the quality of life a subject attributes to this lifespan into one single parameter. Thus, QALYs are subjective and universally applicable outcome parameters for comparing health benefits across sectors, disorders, samples, or populations. It can be assessed in both patients and healthy individuals. Health-related quality of life will be assessed using the KIDSCREEN-10 [57, 58]. In addition, the health service utilization of the participants will be assessed by the “Mannheimer Modul Ressourcenverbrauch” (MRV) [59] and transformed into cost estimates. For this purpose, a catalog of so-called “unit costs” will be compiled for all types of treatments, services, or other healthcare measures that were used.

### Sample size calculation

We assume a small to medium effect (superiority of the two intervention groups over the control condition) on depressive symptomatology at postline after treatment as reported in meta-analyses on interventions for the prevention of depression in C&A [60]. Non-inferiority of the two active intervention groups is assumed. Repeated measures mixed models will be conducted (time*group interaction, alpha = 5%). Assuming a small to medium effect of *f* = 0.13, a lost-to-follow-up rate of 20%, and correlations of 0.2 among repeated measures, *n* = 363 individuals (*n* = 121 per group) need to be recruited for a test of the global hypothesis with 90% power. In case the ANOVA shows significant differences, post-hoc pairwise group comparisons can be conducted at a power of about 80% (Bonferroni correction) with the planned sample size.

Participants will be recruited within the ProHEAD consortium from the school-based sample of *N* = 15,000 screened C&A. Based on prevalence rates of approximately 5–12% of subthreshold depression in adolescents [14], 10% or *n* = 1500 of the screened C&A are expected to be eligible for inclusion in the trial. Thus, in order to meet the required sample size of *n* = 363, about 25% of eligible C&A would have to be willing to participate in the trial.

Criteria for the allocation of participants to the five individual ProHEAD trials are based on latest scientific evidence. However, this is the first time that the overall algorithm is applied on a consortium-wide basis simultaneously screening for various mental health problems. Therefore, an intermediate data analysis will be conducted following completion of 10% of the screening assessments (*N* = 1500) in order to determine the actual allocation ratio to the five ProHEAD trials and to adjust the screening algorithm if necessary.

### Statistical analysis plan

All analyses will be conducted in accordance with intention-to-treat (ITT) principles, i.e. all randomized individuals will be included in the analysis [61]. In addition to the primary ITT analysis, per-protocol analysis will be conducted.

For the primary outcome, repeated measures mixed models (time*group interaction) will be conducted to test for differences in efficacy between the intervention groups. In case of statistically significant results, group differences will be investigated using post-hoc pairwise comparisons (Bonferroni correction). Secondary analyses using repeated measures mixed models will be performed on variables related to help-seeking behavior and depression stigma. In addition, repeated measures mixed models will be conducted to examine long-term group differences in PHQ-A scores at the six-month, one-year, and two-year follow-up timepoints, respectively. All analyses will include testing for interaction effects with study site (time*group*study site interaction).

Additionally, cost-effectiveness analyses and cost-utility analyses will be conducted. Cost-effectiveness analyses include the calculation of the ICER. The ICER indicates the additional cost for each additional (primary) outcome that has to be paid under routine care conditions. During these analyses, standard health economy techniques will be applied, such as bootstrapping techniques for estimating ICER variability, the calculation of cost-effectiveness acceptability curves (CEAC), and calculation of willingness-to-pay (WTP) criteria [62, 63]. In addition, a cost-utility study will be conducted that requires the transformation of longitudinal quality of life data (assessed with the KIDSCREEN-10) into preference measures, for the calculation of QALYs lost or gained during follow-up in order to calculate costs per QALY of the intervention.

Analyses will be performed using IBM SPSS Statistics and *p* values < 0.05 will be considered statistically significant.

### Organization, quality assurance, and data management

Research data will be collected in a pseudonymized manner by means of online questionnaires. Data quality will be ensured by conducting automatic validity and range checks at data entry. The confidentiality of participants is secured by providing unique study identifiers unrelated to the real name. All study-related data will be stored on secure servers at the principal investigator’s institution with frequent back-up procedures in place. Data will be stored for 10 years at the primary research institution. Data handling and access will follow German and European Union legal regulations concerning data protection and data security. The Coordination Center for Clinical Trials (KKS) Heidelberg will monitor study-related procedures at the five recruiting centers. Specifically, the recruitment of schools within the target regions and the recruitment of students within these schools will be monitored in order to ensure adherence to the study manual and documentation guidelines as well as equivalent procedures at all sites. In addition, an independent Data and Safety Monitoring Board (DSMB) as defined in ICH-GCP will assess the progress of the trial, data safety, and the clinical efficacy endpoints.

### Safety reporting

There is no obvious risk for participating C&A. The study does not involve any restriction to standard care. All participants will receive information on where to seek help for mental health problems within the ProHEAD consortium. In case of C&A reporting suicidal plans or suicide attempts, special emergency procedures will be put in place allowing immediate contact of the participant in order to assess risks and refer to appropriate care. Potential serious adverse events (SAE) will be reported to the local ethics committee, the DSMB, and the KKS.

### Dissemination

Trial results will be published in peer-reviewed international journals and will be presented at national and international conferences. Substantial protocol modifications will be communicated to the ethics committee, trial registry, DSMB, and all relevant parties.

## Discussion

This study represents the first RCT investigating efficacy and cost-effectiveness of two online interventions for C&A at risk for depression. To our knowledge, this is the first study, aiming at large scale, sustainable implementation of Internet-based indicated prevention of depression within an integrated infrastructure that aims at health promotion, prevention of clinical symptoms, and facilitating help-seeking. Research on the cost-effectiveness of such interventions is sparse as well and the results of the study will provide a strong basis for decision-making for stakeholders and policy makers in the field. Also, the trial will provide new insights into so far understudied aspects of E-mental health programs, such as potential negative effects of online interventions and user characteristics. Further, this RCT will overcome current shortcomings of available studies, with a particular focus on professional guidance by mental health professionals for participants and longer follow-up periods and provide an internationally consented, guided self-management tool which has been broadly used in adults with milder forms of depression in 11 different languages/countries already.

This RCT will advance the state of the art by bringing an established, guided, online-delivered self-management approach, which is well implemented into routine care, to the large population of unrecognized C&A with subthreshold depression being at risk of developing a depressive disorder.

In summary, the trial will: (1) significantly add to the existing evidence on Internet-based indicated prevention of depression in C&A at risk for the development of depression; (2) be the first study to investigate effects of Internet-based indicated prevention on help-seeking in C&A with subthreshold depressive symptoms; and (3) provide cost-effectiveness data on Internet-based indicated depression prevention.

### Trial status

The trial was registered at the German Register for Clinical Trials (DRKS) under the title “ProHEAD – Promoting Help-seeking using E-technology for Adolescents. Sub-project 4: Efficacy and cost-effectiveness of two online interventions for children and adolescents at risk for depression (E.motion trial)”, identification code: DRKS00014668. International trial-registration took place through the “international clinical trials registry platform” with the secondary ID S-086/2018.

The recruitment period for the trial will start in October 2018 and is predicted to continue until March 2020.

## Additional files

Additional file 1: Standard Protocol Items: Recommendations for Intervention Trials (SPIRIT) Checklist. (DOC 123 kb)

## Abbreviations

AHSQ: Actual Help-Seeking Questionnaire
AUDIT: Alcohol Use Disorders Identification Test
C&A: Children and adolescents
CBT: Cognitive-behavioral therapy
CEAC: Cost-effectiveness acceptability curves
CRAFFT-d: Car, Relax, Alone, Forget, Friends, Trouble
DRKS: German Register for Clinical Trials
DSMB: Data and Safety Monitoring Board
DSM-IV: Diagnostic and Statistical Manual of Mental Disorders, fourth edition
DSS: Depression Stigma Scale
GHSQ: General Help-Seeking Questionnaire
IASMHS: Inventory of Attitudes Toward Seeking Mental Health Services
ICER: Incremental cost-effectiveness ratio
iFD: iFightDepression
IIT: Investigator-initiated trial
INEP: Inventory for the assessment of negative effects of psychotherapy
ITT: Intention-to-treat
KIDSCREEN-10: Health-related quality of life measure for children and adolescents
KKS: Coordination Center for Clinical Trials
MRV: Mannheimer Modul zum Ressourcenverbrauch
PHQ-A: Patient Health Questionnaire-9 modified for Adolescents
QALY: Cost per quality-adjusted life year
RCT: Randomized controlled tial
SAE: Serious adverse event
SDQ: Strengths and Difficulties Questionnaire
SEED: Short Evaluation of Eating Disorders
WCS: Weight Concerns Scale
WTP: Willingness-to-pay
ZUF-8: German version of the Client Satisfaction Questionnaire
